# Analysis and Follow up of Endoscopy Results in 1099 Patients with Terminal Ileum Lesions

**DOI:** 10.1155/2020/8838613

**Published:** 2020-12-04

**Authors:** Qinglian Zhong, Anye Zhang, Jian Huang, Wen Yan, Jiayu Lin, Qun Huang, Xiaoshan Huang, Tao Yu, Luhong Zhu, Chen Xu

**Affiliations:** ^1^Department of Gastroenterology, The Eighth Affiliated Hospital, Sun Yat-sen University, Shenzhen, Guangdong 518033, China; ^2^Sun Yat-sen University·Shenzhen, Shenzhen, Guangdong 518033, China; ^3^University of Delaware, Newark, DE, USA

## Abstract

**Objective:**

We aim to analyze the diagnostic yield, diagnostic accuracy, and delayed diagnosis of patients with terminal ileum lesions, providing follow-up suggestions for suspected patients.

**Methods:**

We carried out an analysis of 1099 patients who had terminal ileum lesions in our hospital from 2009 to 2019. The endoscopy reports and histopathology reports of terminal ileal biopsies were recorded. Clinical diagnosis and management were reviewed to determine whether there was a need to correct after a follow-up endoscopy result.

**Results:**

A total of 1099 patients were found to have terminal ileum lesions, among which 959 in 1099 patients (87.26%) were diagnosed as benign, 17 in 1099 patients (1.55%) were diagnosed as malignant, and 123 in 1099 patients (11.19%) were diagnosed as suspected. The diagnostic accuracies of terminal ileal polyp, cyst, cancer, eosinophilic enteritis, parasite, lymphofollicular hyperplasia, and amyloidosis were 100%. The diagnosis was delayed in 9.93% of Crohn's disease (CD) and 12.5% of lymphoma. Among the definite cases, the diagnosis was corrected during the follow-up in 12.5% of the patients, while the clinical treatment was corrected during the follow-up in 17.86% of the patients. Among the suspected cases, the diagnosis and treatment was corrected in 61.11% of the patients during the follow-up.

**Conclusion:**

Coincident diagnosis of ileitis and ileum ulcer is low. Delayed diagnosis of Crohn's disease and lymphoma were observed in a certain proportion of patients with terminal ileum lesions. A follow-up endoscopy was strongly recommended for these suspected patients with terminal ileum lesions.

## 1. Introduction

The terminal ileum, an important part of the digestive system, is located at the most distant portion of the small intestine. It is the junction of small intestine and colon, where feces are accumulated, as well as the reflux from colon to ileum that could cause bacterial transplantation and translocation [1]. The special anatomical position and physiological characteristics of the terminal ileum may induce immune response that results in mucosal damage [2, 3]. However, the clinical presentation of terminal ileal lesions is similar to colon diseases, such as abdominal pain, diarrhea, hemafecia, anemia, and constipation, resulting that the ileal disease be often ignored or misdiagnosed. In recent years, the detection rate of terminal ileum lesions has been increasing due to the colonoscopy and terminal ileum intubation [4].

During the endoscopy, the presence of the terminal ileum lesions includes erythema, erosions, ulcers, mucosal friability, and granularity [5]. Such terminal ileum lesions may be the results of a wide variety of diseases affecting the terminal ileum. These diseases include lymphoid hyperplasia, lymphoma, radiation enteritis, infections, ulcerative colitis, and Crohn's disease (CD) [1]. Particularly, the terminal ileum is the most frequent localization of Crohn's disease. Recent studies pointed out the importance of early diagnosis of CD and suggest that patients receiving an earlier intensive treatment could have a better clinical outcome [6]. Misdiagnosis of CD may result in patients occasionally receiving an unnecessary operation or having inadequate response to therapy. However, it is still extremely difficult for the differential diagnosis of CD and other terminal ileitis [1, 7, 8]. In light of this, it would be interesting to learn more about terminal ileum lesions, especially for early CD screenings.

In this study, we investigated the terminal ileum lesions detected during endoscopy. We aimed to analyze the diagnostic yield, diagnostic accuracy, and delayed diagnosis of patients with terminal ileum lesions and to evaluate the need for a follow-up endoscopy for suspected cases.

## 2. Patients and Methods

The data were obtained from medical records and computerized endoscopy database in the Eighth Affiliated Hospital, Sun Yat-sen University, between January 2009 and March 2019. The hospital's medical gastroenterology department performed more than 2000 colonoscopies per year. Five endoscopists currently staff the department. The study had been approved by the ethics committee of the University and adhered to the tenets of the Declaration of Helsinki. Additionally, written informed consent was obtained from the patients when they perform endoscopy. We performed an analysis of all patients with terminal ileal lesions who had terminal ileal biopsies during a colonoscopy (Olympus CF-240L, CF-260AZI) or enteroscopy (Fujinon EN-450P5/28). The endoscopic findings and histopathology reports of terminal ileal biopsies were recorded. Clinical diagnosis and management were reviewed to determine whether there was a change after a follow-up endoscopy result.

The main observations of the patients with terminal ileum lesions are as follows: ① disease spectrum; ② accuracy of diagnosis; ③ delayed diagnosis and management change; and ④follow-up suggestions of terminal ileum lesions according to the results of follow-up.

Statistical analysis was performed using SPSS 19.0 for Windows (SPSS Inc. Chicago, IL). Numerical data were expressed as mean ± standard deviation. Categorical variables were analyzed by the Chi-square test. A *p* value <0.05 was considered to be statistically significant.

## 3. Results

### 3.1. Clinical Characteristics of Patients with Terminal Ileum Lesions

A total of 1099 patients were found to have terminal ileal diseases, and 602 patients were male, while 497 patients were female, with an average age of 42.48 ± 16.5 years old (13–94 years old). The indications for endoscopy were abdominal pain in 405 patients (36.85%), diarrhea in 297 patients (27.02%), constipation in 70 patients (6.37%), and both in 28 patients (2.55%). Other indications were bleeding or anemia (*n* = 38, 3.46%), abdominal mass (*n* = 10, 0.91%), and weight loss (*n* = 8, 0.73%), etc. There were 268 asymptomatic patients (24.39%) (Table 1).

Endoscopic features of ileum lesions were nonspecific and often presented as ileitis (such as hyperemia, edema, erythema, and erosion), ulcer, hyperplasia, mass, stenosis and polyp, etc. Ileitis and ileum ulcer were mainly endoscopic findings both in established or suspected cases (Table 2).

Combined with endoscopic and pathological findings, there were 959 patients (87.26%) diagnosed as benign, including 476 patients of ileitis (43.31%), 249 patients of ileal ulcer (22.45%), 127 patients of CD (11.56%), 69 patients of lymphofollicular hyperplasia (6.28%), 12 patients of tuberculosis (1.09%), 9 patients of polyps (0.82%), 8 patients of eosinophilic enteritis (0.73%), 3 patients of cyst (0.27%), 2 patients of Behcet's disease (0.18%), 2 patients of amyloidosis (0.18%), 1 patient of intestinal lymphangiectasia (0.09%), and 1 patient of parasites (0.09%). There were 17 patients of malignant lesions (1.55%), including 14 patients of lymphoma (1.27%) and 3 patients of cancer (2 patients of adenocarcinoma and 1 patient of small round cell tumor (2.27%). There were 123 patients (11.19%) of suspected patients with terminal ileal lesions, most were ileitis and ulcerative lesions, in which the clinical diagnosis was not clear yet (Table 3).

### 3.2. Follow-Up of Patients with Terminal Ileum Lesions

**Figure 1:**
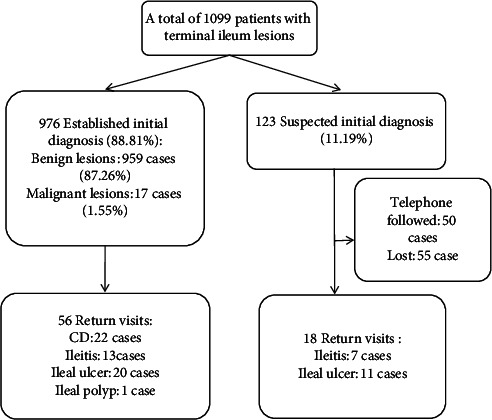
Flow chart of patients with terminal ileum lesions.

**Figure 2:**
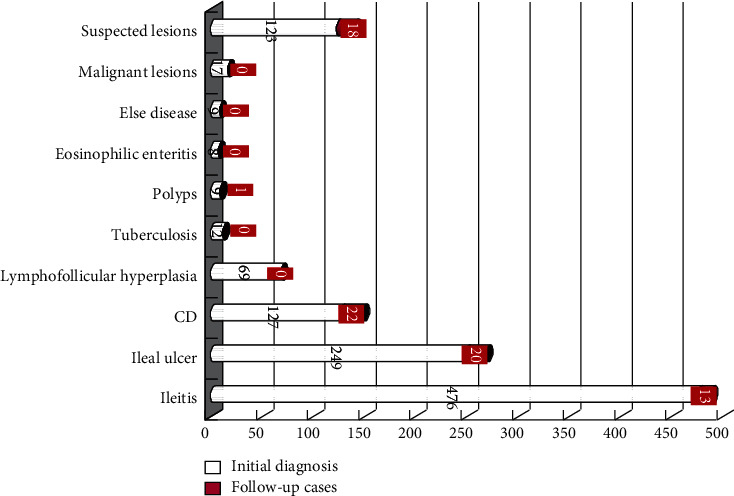
Follow-up of patients with terminal ileum lesions (case).

The flow chart of follow-up is summarized in Figure 1. There were 74 patients returned to our hospital for follow-up endoscopy and biopsy diagnosis. Most of the patients with benign terminal ileum lesions did not have any return visit. CD had a higher proportion of follow-up (22/127, 17.32%), followed by ileal ulcer (20/249, 8.03%) and ileitis (13/476, 2.73%) (Figure 2). Among 123 suspected patients, 50 patients were followed up by telephone, 21 patients without pathological results, and 29 patients without second colonoscopy. Another 55 patients were lost.

The diagnosis and management are summarized in Table 4. The diagnosis was corrected in 18 of 74 patients (24.32%) with ileitis or ileal ulcer. Among the 56 return patients with established initial diagnosis by endoscopy at the first time, 7 of 56 patients (12.5%) were diagnosed differently in follow-up and 10 of 56 patients (17.86%) changed the treatment. 3 patients with CD were found to be complicating intestinal tuberculosis during follow-up. Among the 18 return patients with suspected diagnosis, 11 of 18 patients (61.11%) alter the diagnosis and management after second check which is a surprisingly high ratio. One interesting case is an ileal ulcer patient who did not heal after 6-year tracked observation.

### 3.3. Accuracy and Delay Rate in Diagnosis of Terminal Ileum Lesions

The final diagnosis was reached based on the follow-up of terminal ileum lesions, symptoms, pathological results, and endoscopy findings. Our results showed that the accuracy was 100% for terminal ileal polyps, cysts, eosinophilic enteritis, parasites, lymphofollicular hyperplasia, amyloidosis, and cancer. There were 14 patients with delayed CD diagnosis (14 of 141 patients, 9.93%) and 2 patients with delayed lymphoma diagnosis (2 of 16 patients, 12.5%). However, the ileal ulcer had a poor coincidence rate.

### 3.4. Follow-Up Time of Patients with Terminal Ileum Lesions

The review time of the 74 patients ranged from 1.03 to 108.5 months, with an average of 21.92 ± 20.71 months. The mean delayed time of final diagnosis was 28.36 ± 25.3 months in 14 patients with CD, 22.37 ± 16.37 months in 2 patients with lymphoma, respectively.

## 4. Discussion

Our study shows that most terminal ileum lesions are benign, and a few are malignant. Mid-aged group has a higher incidence of terminal ileum lesions. Our results are consistent with the findings of several previous studies [9–11]. Combined with the endoscopic findings and pathological results, we achieved a high diagnostic accuracy in some ileum disease. The coincidence rates of ileum cysts, amyloidosis, eosinophilic enteritis, lymphofollicular hyperplasia, parasites, polyps, and cancer were 100%. Our results suggest that these 7 kinds of diseases can be diagnosed effectively, thanks to their specific pathological characteristics. However, the ileitis and ileal ulcer are nonspecific in pathological results. Thus, it is difficult to diagnose and distinguish in the early stage of many diseases that affect terminal ileum, such as intestinal tuberculosis, CD, and lymphoma. Therefore, the coincident diagnosis of ileitis and ileum ulcer is low.

Our study reveals that 24.32% of patients with ileitis and ileal ulcer developed into lymphoma and CD during follow-up. Some literature studies suggest it is not cost-effective to carry out ileoscopy on all patients [11–15] for reasons that ileitis and ileal ulcer are rarely developed to malignant diseases and their detection rate is low. However, from 2008 to 2016, literature review analyzed that the development of CD in the follow-up of ileitis patients ranged from 1.07% to 34.5% [16–21]. With the extension of follow-up time, the proportion of CD increased. Thus, our study suggests that follow-up should be encouraged on some selected patients, especially for those with isolated terminal ileal ulcer of unknown etiology, right lower abdominal pain, anemia, diarrhea, or clinically suspected CD [9–11, 15, 22, 23].

The early diagnosis of CD patients plays a critical role in the management of CD. In 2010, Peyrin-Biroulet [6] proposed a definition of early CD: the CD patient has a duration less than 2 years and no gastrointestinal injury and dysfunction. Meantime, they put forward a concept of “therapeutic window of opportunity.” It is believed that the earlier the intensive treatment for CD patients, the better to control the disease, injury, and disability. In 2011, international experts agreed to update the Paris definition of early CD based on the duration of disease after diagnosis (≤18 months) and without immunosuppressant or biological agents [24]. However, the exact timing of early diagnosis and treatment of CD has not been determined yet. Delayed diagnosis is still a problem for both CD patient and doctor, indicating the loss of the therapeutic window. In an IBD cohort in Switzerland, 75% of CD patients were diagnosed within 24 months [25]. A questionnaire survey of IBD patients in eastern China shows that only 33.0% of patients were diagnosed at the first visit [26]. In our cohort, 9.93% of CD was delayed in diagnosis at least, but in view of the excluded numbers of suspected cases, the proportion may be larger. Therefore, it is necessary to have follow-up ileal intubation less than 2 years to screen the early CD and get the final definition.

The reasons for delayed diagnosis are as follows: firstly, the clinical manifestations, endoscopic findings, and pathological features are not specific in early CD. Secondly, CD processed slowly over several years. Thirdly, of the 1099 patients with terminal lesions, 24.39% were asymptomatic and 87.26% were benign. Therefore, the follow-up visit was ignored or delayed.

Facing the difficulty in diagnosis of terminal ileitis, including CD and lymphoma, management algorithm for these patients with terminal ileum lesions is required. Besides the endoscopic and histopathological results of the individuals, their clinic characteristics, laboratory results, and imaging results are also crucial to diagnose and distinguish the disease [1]. It is also noted that there was individual difference in the patients and disease course in clinical practice, and an individual algorithm based on clinical, laboratory, endoscopic, and histopathological evaluation of patients is encouraged.

In addition, our study showed there are 12.74% of ileum malignant lesions and suspected lesions. Thus, the endoscopists should not only look for colon diseases but also check the terminal ileum regularly to reduce the missing diagnosis. We also observed that there could be further development of the ileal diseases found in the second endoscopic biopsy, which leads to the correction of diagnosis and management. Thus, we strongly recommend a follow-up for ileal uncertain lesions in order to avoid the delayed diagnosis.

There are some limitations of this study. Some undiagnosed cases with nonspecific ileal lesions still need longer follow-up, which may affect the accuracy of the data slightly. We will extend the follow-up time and continue to observe the changes in the condition of these patients.

In conclusion, the coincident diagnosis of ileitis and ileum ulcer is low. Delayed diagnosis of CD and lymphoma were observed in a certain proportion of patients with terminal ileum lesions. A follow-up endoscopy was strongly recommended for these suspected patients with terminal ileum lesions.

## Figures and Tables

**Table 1:** Clinical characteristics of patients with terminal ileum lesion.

	Number of patients	Percentage (%)
Gender		
Male	602	54.77
Female	497	45.23

Age		
<16 yr	37	3.37
16∼40 yr	497	45.22
41∼59 yr	387	35.21
>60 yr	178	16.20

Indication		
Abdominal pain	405	36.85
Diarrhea	297	27.02
Constipation	70	6.37
Bleeding or anemia	38	3.46
Abdominal mass	10	0.91
Weight loss	8	0.73
Fever	5	0.45
Other	26	2.37
Asymptomatic	268	24.39

**Table 2:** Endoscopic findings of patients with terminal ileum lesion.

Endoscopic feature	Established case (*n*, %)	Suspected case (*n*, %)	Total (*n*, %)	*p*
*N*	976	123	1099	<0.01
Hyperemia, edema, erythema, and erosion	557 (57.07%)	119 (96.75%)	676 (61.51%)	<0.01
Ulcer	299 (30.63%)	74 (60.16%)	373 (33.93%)	<0.01
Hyperplasia	69 (7.07%)	0 (0.00%)	69 (6.28%)	>0.05
Mass	17 (1.74%)	0 (0.00%)	17 (1.55%)	>0.05
Stenosis	20 (2.05%)	2 (1.63%)	22 (2.00%)	>0.05
Polyp	9 (9.22%)	0 (0.00%)	9 (0.82%)	>0.05
Others	5 (0.51%)	0 (0.00%)	5 (0.45%)	<0.01

**Table 3:** Disease spectra of patients with terminal ileum lesion.

	Number of patients	Percentage (%)
Benign	959	87.26
Ileitis	476	43.31
Ileal ulcer	249	22.45
CD	127	11.56
Lymphofollicular hyperplasia	69	6.28
Tuberculosis	12	1.09
Polyps	9	0.82
Eosinophilic enteritis	8	0.73
Cyst	3	0.27
Behcet's disease	2	0.18
Amyloidosis	2	0.18
Intestinal lymphangiodilation	1	0.09
Parasites	1	0.09

Malignant	17	1.55
Lymphoma	14	1.27
Adenocarcinoma	2	0.18
Small round cell tumor	1	0.09
Suspected	123	11.19

**Table 4:** Follow-up of patients with terminal ileum lesions.

	Initial diagnosis (case)	Diagnosis after follow-up (case)	Time of follow-up (months)	Management change
Initial diagnosis	56			
	CD: 22	Complicating intestinal tuberculosis: 3	(23.3 ± 16.1)	Treatment change
	Ileitis: 13	Intestinal tuberculosis: 1 CD: 1 Behcet's disease: 1	(17.7 ± 22.1)	Treatment change
	Ileal ulcer: 20	Lymphoma: 1 CD: 3	(21.9 ± 22.2)	Treatment change
	Polyp: 1	Polyp: 1	12	No change

Suspected cases	18			
	Ileitis: 3	Ileitis: 3	(11.85 ± 6.38)	No change
	Ileitis: 4			
	Ileal ulcer: 6	CD: 10	(27.7 ± 29.1)	Treatment change
	Ileal ulcer: 1	Lymphoma: 1	6	Treatment change
	Ileal ulcer: 4	Ileal ulcer: 4	(33.7 ± 14.2)	Further follow-up

## Data Availability

The data used to support the findings of this study are available from the corresponding author upon request.
